# INTERDISCIPLINARY PAIN REHABILITATION FOR PATIENTS WITH EHLERS-DANLOS SYNDROME AND HYPERMOBILITY SPECTRUM DISORDERS

**DOI:** 10.2340/jrm.v56.12431

**Published:** 2024-02-07

**Authors:** Peter MOLANDER, Mehmed NOVO, Åsa RINGQVIST, Andrea HÅLLSTAM, Hugo HESSER, Monika LÖFGREN, Britt-Marie STÅLNACKE, Björn GERDLE

**Affiliations:** 1 Pain and Rehabilitation Centre and Department of Medical and Health Sciences; 2 Department of Behavioural Sciences and Learning, Linköping University, Linköping; 3 Department of Community Medicine and Rehabilitation, Rehabilitation Medicine, Umeå University, Umeå; 4 Department of Neurosurgery and Pain Rehabilitation, Skåne University Hospital, Lund; 5 Department of Clinical Sciences, Danderyd Hospital, Karolinska Institutet, Stockholm; 6 School of Behavioural, Social and Legal Sciences, Center for Health and Medical Psychology, Örebro University, Sweden

**Keywords:** chronic pain, rehabilitation, Ehlers-Danlos syndrome

## Abstract

**Objective:**

Chronic pain is a common manifestation of Ehlers-Danlos syndrome and hypermobility spectrum disorders; thus it is often suggested that patients undergo generic interdisciplinary pain rehabilitation, despite there being little evidence to support this decision. The aim of this study is to examine the effectiveness of standard rehabilitation programmes for chronic pain on patients with Ehlers-Danlos syndrome and hypermobility spectrum disorders, compared with patients with other chronic pain disorders.

**Subjects:**

Data, collected between 2008 and 2016, were extracted from a Swedish national registry. The patient data comprised of 406 cases with Ehlers-Danlos syndrome or hypermobility spectrum disorders, 784 cases with a whiplash-related diagnosis, 3713 cases with diagnoses relating to spinal pain, and 2880 cases of fibromyalgia.

**Methods:**

The differences between groups on key outcome measures from pre- to 1-year follow-up after interdisciplinary pain rehabilitation were analysed using linear mixed effects models. Sensitivity analysis in the form of pattern-mixture modelling was conducted to discern the impact of missing data.

**Results:**

No significant differences were found in improvements from pre- to 1-year follow-up for patients with Ehlers-Danlos syndrome or hypermobility spectrum disorder compared with other diagnostic groups regarding measures of health-related quality of life, mental health, or fatigue. At follow-up, differences in pain interference (d = –0.34 (95% confidence interval [95% CI] –0.5 to –0.18)), average pain (d = 0.22 (95% CI 0.11–0.62)) and physical functioning (d = 2.19 (95% CI 1.61–2.77)) were detected for the group with spinal-related diagnoses in relation to those with EDS/HSD, largely due to pre-treatment group differences. Sensitivity analysis found little evidence for missing data influencing the results.

**Conclusion:**

This study suggests that patients with Ehlers-Danlos syndrome/hypermobility spectrum disorders may benefit from inclusion in an interdisciplinary pain rehabilitation programme.

LAY ABSTRACT

People with hypermobility often develop chronic pain. Such patients may be offered rehabilitation programmes that aim to reduce pain and increase functioning, but which have often been developed for people with chronic pain conditions that do not necessarily involve hypermobility. No previous study has examined whether these types of rehabilitation programme are beneficial for people with hypermobility. In this study, we compared the effects of ordinary pain rehabilitation programmes for people with hypermobility against other common pain conditions. The results of this study suggest that people with hypermobility benefit from these programmes, and that the gains are often similar to those for patients with more common chronic pain conditions.

Musculoskeletal disorders are common reasons for seeking healthcare (1). This group includes patients with joint hypermobility, which can be caused by conditions such as hypermobility spectrum disorders (HSD), which, prior to 2017, were known as hypermobility syndrome (HMS). Another common example is Ehlers-Danlos Syndrome (EDS). In addition to pain and disability, the symptoms of these conditions are difficult for clinicians to interpret.

EDS is a highly heritable family of conditions, comprising 13 types, signified by collagen deficiencies, resulting in multiple medical problems, such as skin hyperextensibility, cardiovascular abnormalities, dysautonomia and structural and functional gastrointestinal abnormalities (2). The most common type is called hypermobile EDS (3). HSD, on the other hand, are characterized by generalized joint hypermobility that does not meet the criteria for systemic problems connected to the collagen defects found in EDS (4). Significant in both conditions are negative consequences that may start in childhood, followed by extensive problems in adulthood (e.g. difficulties in being occupationally active or carrying out household work) (5). Chronic pain in EDS and HSD includes different components of nociceptive, neuropathic and nociplastic mechanisms (6), and thus the pain is generally complex and often generalized. Quality of life is often affected, with poor physical, psychosocial, social participation, and overall functioning (5, 7), leading to demands on healthcare services and requests for rehabilitation, e.g. interdisciplinary pain rehabilitation programmes (IPRPs) (8).

In a large recent study based on the Swedish National Quality Registry for Pain Rehabilitation (SQRP), we examined patient-reported symptoms and problems of EDS and HSD, which showed congruence to a large degree (8). Therefore, we treated them a single group. We also compared EDS/HSD with other common pain conditions (fibromyalgia, whiplash-associated disorders (WAD) and spinal pain) (8). Young age and long history of pain were the characteristic traits of the EDS/HSD group. While reported levels of anxiety and depressive symptoms were similar in all groups, patients with fibromyalgia reported the highest levels of pain intensity. Both patients with EDS/HSD and patients with fibromyalgia reported lower vitality and physical health compared with other groups investigated in that study with either spinal- or neck-related pain. As the SQRP is a large registry, which may contain variability between sites in certain diagnoses, there is a possibility that patients with overlapping symptoms, such as the many locations of pain found in both EDS and fibromyalgia, will have different main diagnosis that could confound the results, since diagnostic validation is not possible. In summary, a broad impact of chronic pain on daily life was demonstrated in patients with EDS/HSD and fibromyalgia, implying a prerequisite for complex interventions, such as IPRPs.

As there is no curative treatment available for chronic pain (6), the goal for patients is to manage pain and related symptoms, such as fatigue, insomnia, and emotional distress (9). Although these phenomena are very commonly viewed as a consequence of pain, the relationship is often bidirectional, meaning that they also can cause chronic pain (10). Furthermore, a low quality of life, lack of social participation and problems with occupational activity are well documented (7). In complex pain conditions, IPRPs, based on the bio-psycho-social model (11), are recommended and favoured by evidence (12). In IPRPs, a team including different healthcare professionals (e.g. physicians, physiotherapists, psychologists, occupational therapists, nurses, and social workers) plan and co-ordinate rehabilitation interventions with aims that are defined together with the patient. The programmes usually include patient education, physical activity, work-related activities, and coping strategies. These are tailored to the patient, meaning that interventions vary depending on individual needs, such as different forms of exercise and physiotherapy. The interventions are based on cognitive behavioural therapy theories and are usually performed in groups over several weeks (13, 14). In diagnoses such as fibromyalgia, chronic back pain, and WAD, IPRPs have been shown to decrease pain, and increase quality of life, physical functioning and return to work compared with unimodal treatments (15, 16). A recent practice-based evidence study of patients with chronic pain who had different non-malignant diagnoses included in the SQRP showed improvements after IPRPs and at the 1-year follow-up in several important outcomes (e.g. pain, pain interference, vitality, health-related quality of life). The most significant improvements were observed for patients with the worst self-reported clinical presentation pre-IPRPs (9).

There is a lack of published studies regarding the efficacy and effects of various treatment methods for EDS/HSD, and the evidence level is generally weak (17, 18). The complexity of these conditions makes IPRPs a preferred treatment alternative (19, 20). However, only a few studies have investigated the outcomes of IPRPs for patients with EDS/HSD (21–23). Moreover, there are no studies comparing the outcomes for IPRPs in patients with EDS/HSD with those in patients with other common chronic pain diagnoses. Therefore, it is unclear whether patients with EDS/HSD benefit from general IPRPs in specialist care for patients with chronic pain.

The aim of this study was therefore to address these knowledge gaps, by comparing the outcomes of IPRPs in 4 different diagnostic groups of patients with chronic pain referred to specialist clinics, including a group of patients with EDS/HSD.

## METHODS

### Subjects

Patients who participated in IPRPs and participated in the SQRP from 2008 to 2016 were included. As there were no major differences between EDS and HSD in this dataset, as described in an earlier publication (8), the patients with these diagnoses were combined into a single group: EDS/HSD. Description of the International Classification of Diseases, Swedish version (ICD-10-SE) diagnoses that constituted the other diagnostic groups (WAD, spinal pain (spinal), and fibromyalgia (FMS)) is also available in the earlier publication (8). It is important to note that only the diagnostic code was available in the current study, thereby eliminating the possibility to report on subtypes of the EDS or HSD diagnosis, since ICD-10 contains only 1 code for each condition. Furthermore, due to the nature of the current study, which is based on registry data, the validation of diagnoses was not possible. However, as hypermobile EDS is the most common type, and is quite likely to have chronic pain as a major presenting symptom, it is reasonable to assume that this group represents the majority of patients in the current sample. To investigate differences in outcomes, a set of measures included in the SQRP were chosen that were largely in line with recommendations on pain research outcome measures (24, 25), focusing on pain levels, mental health aspects, physical functioning, and quality of life.

The study was conducted in accordance with the principles of the Declaration of Helsinki and Good Clinical Practice and was approved by the Ethical Review Board in Linköping (Dnr: 2015/108-31).

### Measures

Pain intensity over the previous 7 days was registered using a numerical rating scale (NRS), NRS-7days, which ranges from 0 (no pain) to 10 (worst possible pain) (26). Another aspect of pain measured in the current study was the Pain Interference subscale from the Swedish translation of the West Haven-Yale Multidimensional Pain Inventory (MPI) (27), which assesses the individual’s perceptions regarding how pain interferes with their lives. Furthermore, quality of life was estimated using the European Quality of Life instrument EQ-5D-3L (28). This instrument consists of 2 parts, 1 of which is a thermometer-like scale from 0 to 100 measuring self-rated health state (EQ-VAS). Lastly, 3 subscales of the 36-Item Short Form Health Survey (SF-36) were included (29) (i.e. Physical Functioning (SF36-PF), Mental Health (SF36-MH), and Vitality (SF36-VT)). A more thorough description of the included measures is available in Ringqvist et al. (9).

### Statistical analysis

To determine potential differences in changes from pre- to follow-up assessments between the diagnostic groups, a series of linear mixed effects models (i.e. growth models) were conducted. All growth models included fixed terms for diagnostic group (i.e. 3 dummy-coded variables using EDS as the reference category), time (coded in months from pre-treatment), and time by diagnostic group interaction effects. Visual inspections of observed means for each group and individual trajectories suggested a non-linear change over assessment points. To linearize the relationship of the observed scores over time, a square root transformation of time was used, as suggested by Hedeker & Gibbons (30). Correlated random effects, subject-specific intercepts, and time trends were included in the model, in order to handle dependence among repeated observations over time. The EDS/HSD group was used as a reference category and the other diagnostic groups were compared with that group. Satterthwaite’s approximation for denominator degrees of freedom was used for inferential tests. Effect sizes in the form of standardized mean differences (d) at endpoints, with confidence intervals, were computed, based on model-implied means and formulas provided by Feingold (31). Effect sizes of 0.20–0.49 were considered small, 0.50–0.79 moderate, and ≥ 0.80 large.

Growth models were fitted using full information maximum likelihood estimation and included all individuals with at least 1 valid measurement on the dependent variable in the model. Given a substantial amount of missing data at follow-up assessments, a sensitivity analysis was conducted in the form of a pattern-mixture model, as described by Hedeker & Gibbons (32), to determine whether missing data influenced the findings. In this approach, participants with missing data at post- and/or 1-year follow-up assessments were included in a missing data group (dropouts) and compared with the rest of the participants (i.e. completers). The dummy-coded missing data variable (1 = dropouts, 0 = completers) was entered into the growth model as a main effect and as interactions with other model variables (i.e. diagnostic groups and time). The primary aim was to test whether the 3-way interactions between diagnostic groups, time and missing data variable were statistically significant, as this would indicate that missing data differentially influenced how diagnostic groups changed over time. If significant, new overall averaged endpoint differences between diagnostic groups were computed (i.e. averaging over missing data patterns) derived from the model parameters to determine how missing data influenced the results (see Hedeker & Gibbons (32)). These analyses were performed using LME4-package for R (33).

## RESULTS

**Fig. 1:**
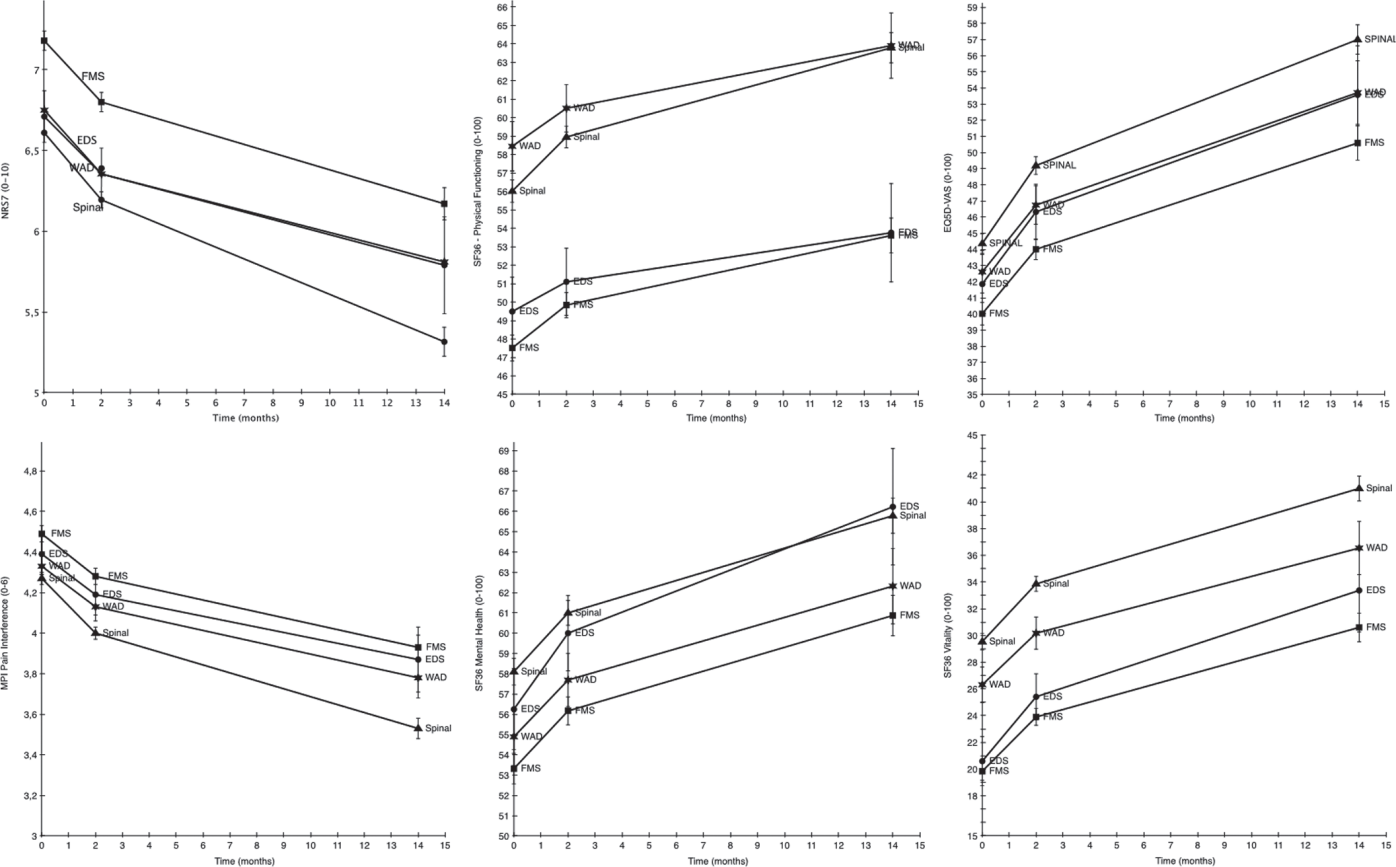
Estimated means (with error bars representing standard errors) for the 6 outcome measures in the 4 diagnostic groups. Note: Ehlers-Danlos syndrome/hypermobility spectrum disorder is abbreviated EDS in this figure. The y-axis is broken on all measures to illustrate group differences. WAD: whiplash-associated disorders; EDS/HSD: Ehlers-danlos syndrome/Hypermobility spectrum disorder; WAD: whiplash-associated disorders;Spinal: Spinal pain; FMS: fibromyalgia. SF36: 36-Item Short Form Health Survey Physical Functioning subscale; EQ5D-VAS: European Quality of Life instrument, visual analogue scale. MPI Pain-Interfer: Multidimensional Pain Inventory, Pain interference subscale. SF36: 36-Item Short Form Health Survey Mental Health subscale. SF36: 36-Item Short Form Health Survey Vitality subscale.

Outcome measure results (means and standard deviations (SD)) are shown in Table I, and have been described in more detail in a previous publication on the same dataset (8). The results of the linear mixed effects models are described below and visualized in Fig. 1.

**Table I:** Outcome measurements for the 4 diagnostic groups at baseline (T1), post-rehabilitation (T2), and 1-year follow-up (T3)

Measures, mean (SD)	EDS/HSD	WAD	Spinal	FMS
NRS-7days T1	6.8 (1.59)	6.83 (1.72)	6.79 (1.72)	7.32 (1.54)
NRS-7days T2	6.16 (1.92)	6.16 (2.01)	5.71 (2.12)	6.49 (1.86)
NRS-7days T3	6.01 (2.07)	5.87 (2.24)	5.54 (2.34)	6.40 (2.01)
SF36-MH T1	55.26 (20.70)	53.65 (21.76)	56.58 (21.19)	51.68 (21.15)
SF36-MH T2	62.42 (20.34)	59.75 (21.53)	64.88 (21.25)	60.11 (21.28)
SF36-MH T3	64.98 (20.06)	61.46 (22.15)	64.75 (21.97)	59.02 (22.11)
SF36-VT T1	18.26 (16.43)	24.45 (18.57)	26.81 (19.04)	17.13 (15.84)
SF36-VT T2	30.69 (21.33)	33.78 (21.78)	39.71 (23.22)	29.39 (21.06)
SF36-VT T3	29.29 (20.91)	34.49 (22.20)	37.69 (24.26)	26.30 (20.93)
SF36-PF T1	49.18 (21.10)	57.95 (19.51)	55.18 (19.83)	46.96 (19.51)
SF36-PF T2	51.69 (19.95)	61.38 (18.83)	61.40 (19.89)	51.31 (19.87)
SF36-PF T3	54.46 (21.93)	64.63 (20.11)	63.29 (21.54)	53.21 (20.92)
MPI Pain-Interfer T1	4.42 (0.98)	4.38 (1.04)	4.33 (1.01)	4.56 (0.92)
MPI Pain-Interfer T2	4.11 (1.01)	4.01 (1.13)	3.84 (1.18)	4.12 (1.07)
MPI Pain-Interfer T3	3.93 (1.14)	3.8 (1.35)	3.58 (1.38)	4.04 (1.17)
EQ5D-VAS T1	40.16 (18.62)	41.38 (19.9)	42.50 (19.02)	38.14 (18.63)
EQ5D-VAS T2	50.26 (20.72)	49.52 (21.19)	53.44 (20.79)	47.89 (21.31)
EQ5D-VAS T3	50.87 (21.90)	53.14 (22.49)	55.16 (22.69)	48.01 (22.16)

SF36: 36-Item Short Form Health Survey; PF: Physical Functioning; MH: Mental Health; VT: Vitality; MPI Pain-Interfer: Multidimensional Pain Inventory, Pain interference subscale; EQ5D-VAS: European Quality of Life instrument, visual analogue scale; WAD: whiplash-associated disorders; EDS/HSD: Ehlers-danlos syndrome/Hypermobility spectrum disorder; WAD: whiplash-associated disorders;Spinal: Spinal pain; FMS: fibromyalgia; SD: standard deviation.

### Response rate

As the registry does not have a formal indicator variable for missingness, the first single patient-reported outcome measure (PROM) item, NRS-7days, was used as a proxy for if the patient had answered the SQRP at the 3 different time-points. These time-points are represented by the responses collected before the IPRP (T1), directly after the IPRP (T2), and at 1-year follow-up (T3). Although the time to complete IPRPs varies between clinics (13), this was set at 2 months post-baseline as a reasonable estimate. The results are shown in Table II.

**Table II:** Responses to the questionnaires of the Swedish Quality Register for Pain rehabilitation (SQRP) per diagnostic group

Timepoint	EDS/HSD	WAD	Spinal	FMS
T1 (0 months)	406 (100%)	784 (100%)	3713 (100%)	2880 (100%)
T2 (2 months)	334 (82%)	678 (87%)	3200 (86%)	2446 (85%)
T3 (14 months)	161 (40%)	431 (55%)	2046 (55%)	1457 (51%)

SQRP: Swedish Quality Register for Pain rehabilitation; EDS: Ehlers-Danlos syndrome; HDS: hypermobility spectrum disorder; WAD: whiplash-associated disorders; FMS: fibromyalgia; T1: baseline; T2: post-rehabilitation; T3: 1-year follow-up.

Response rates are calculated with T1 answers as baseline for patients selected for interdisciplinary pain rehabilitation programmes (IPRPs), it is possible that a small number of patients had unaccounted missing data for that time-point.

### Pain intensity

Overall, all 4 groups improved over time (EDS/HSD, Fibromyalgia, WAD, Spinal) (*B* = –0.25, SE = 0.05, *p* < 0.001), as shown in Fig. 1. When comparing the effects of treatment on pain intensity, i.e. NRS-7days, 1 statistically significant interaction was found, between diagnostic group and time. The Spinal group improved more than the EDS/HSD group (*B* = –0.1, SE = 0.005, *p* = 0.03), i.e the Spinal group scored 0.1 less on NRS-7days per unit of time. This corresponds to an effect size of Cohen’s d = 0.22 (95% CI 0.11–0.62) (i.e. a small effect size) at the final measurement point. It is also noteworthy that the FMS group had a higher pain rating before the IPRP (0.47 points, *p* < 0.001).

### Mental health

Fig. 1 illustrates the overall effect of time (*B* = 0.67, SE = 0.41, *p* < 0.001), with all groups (EDS/HSD, Fibromyalgia, WAD, Spinal) improving over time. The SF36-MH showed 1 significant difference between groups at baseline; the FMS group had a lower baseline value than the EDS/HSD group (*B* = 2.92, SE = 1.09, *p* < 0.001). There were no statistically significant interaction effects between groups and time.

### Vitality

On the SF36-VT, there was an effect of time (EDS/HSD, Fibromyalgia, WAD, Spinal) (*B* = 3.42, SE = 0.47, *p* < 0.001). There were also 2 significant baseline differences (Fig. 1). The WAD group scored higher at baseline than the EDS/HSD group (*B* = 5.72, SE = 1.16, *p* < 0.0001). There was an even greater difference at baseline between the EDS/HSD group and the Spinal group, (*B* = 8.92, SE = 0.99, *p* < 0.0001). There were no statistically significant interaction effects between group and time.

### Physical functioning

For the SF36-PF, all groups improved over time (Fig. 1) (*B* = 1.14, SE = 0.04, *p* = 0.001). There was also a significant difference between the EDS/HSD and WAD groups in their baseline values (*B* = 8.96, SE = 1.17, *p* < 0.0001), and between the EDS/HSD and Spinal groups (*B* = 6.53, SE = 1.00, *p* < 0.0001) at baseline. Furthermore, there was a significant interaction effect between group and time when comparing the EDS/HSD group with the Spinal group, (*B* = 0.94, SE = 0.36, *p* < 0.001). The standardized mean difference between these groups at the last time-point was *d* = 2.19 (95% CI 1.61–2.77), which was interpreted as a large effect. However, this difference in change should not be overstated, as the diagnostic groups differed substantially before rehabilitation began (Fig. 1).

### Pain interference

There was a significant effect of time (*B* = –0.14, SE = 0.02, *p* < 0.001) (Fig. 1). There was also a significant difference in baseline values on MPI-pain-interference; the Spinal group scored lower than the EDS/HSD group (*B* = –0.12, SE = 0.05, *p* = 0.02). There was also a difference between these groups in their endpoint ratings; the Spinal group rated a higher improvement (*B* = –0.06, SE = 0.02, *p* < 0.01) with a small size effect, *d* = –0.34 (95% CI –0.5 to –0.18).

### Health-related quality of life

On the EQ-VAS, all 4 diagnostic groups rated a higher health perception 1 year after treatment ended (Fig. 1) (*B* = 3.17, SE = 0.05, *p* < 0.001) than at baseline. The Spinal group scored significantly higher on their baseline rating (*B* = 2.51, SE = 0.99, *p* = 0.01) than the EDS/HSD group. There were no statistically significant interaction effects between group and time.

### Effect of missing data on the primary results

When analysing the effects of missingness on the results, using a pattern-mixture model, no significant interactions were found between diagnostic group, time and pattern of missing data for the variables NRS-7days, EQ-VAS, SF36-MH, SF36-VT (all *p* > 0.07). However, a significant 3-way interaction effect was found for SF36-PF for the EDS/HSD group in comparison with the other 3 diagnostic groups (all *p* < 0.01), indicating that indviduals with missing data at the follow-up time-point had a different response over time depending on the pain group. These differences are shown in Table SI. There was also a significant 3-way interaction on MPI-Pain-interfer for the comparison between the EDS/HSD and FMS groups (*B* = –0.57, *p* = 0.003).

To determine how this influenced the findings, new population parameter estimates of endpoints that took missing data patterns into account were computed using proportion-weighted estimates across subgroups with different missing data patterns. Only small differences were observed on means and associated standard errors between the original analysis and the pattern mixture model, indicating that the results were still robust to potential violations of the missing data assumption (i.e. Missing at Random (MAR)). Taken together, results from the sensitivity analyses indicate that missing data had no substantial impact on the main findings and that missing data did not bias the key findings obtained in analyses based on the MAR assumption.

## DISCUSSION

This study compared the effects of IPRPs in patients with a hypermobility-related disorder (EDS/HSD) in comparison with patients in other common diagnostic groups. While the included diagnostic groups at times differ somewhat in their health-ratings at the start of rehabilitation, the overall impression is that the gains made are largely comparable regardless of main diagnosis, with a few exceptions discussed below. These improvements at the 1-year follow-up included a main effect of time for all measures. Furthermore, the effect of missing data was examined. Using a pattern-mixture model (32), it was concluded that missing data biasing the results was not a major concern for this dataset.

Although there are differences between diagnostic groups, they were not that different in baseline values and in gains made at the 1-year follow-up. There are 2 possible explanations for this. First, only patients who have both a high complexity of chronic pain that affects daily life and display motivation for behavioural change are invited to participate in IPRPs, meaning that there are similarities not related to the main diagnosis. Secondly, chronic pain is now more recognized as a disease in its own right (34). In this view, the pain diagnosis might not be a decisive prognostic factor. Thus, it is reasonable that different pain conditions may have similar trajectories, at least for many subtypes.

Although patients with EDS/HSD had comparable gains from rehabilitation to other diagnostic groups on most outcome domains, a few notable exceptions were detected compared with the group with a spinal diagnosis. These differences were detected on measures of physical functioning (SF36-PF), and on the 2 measures of pain aspects (NRS-7days and MPI-Pain-interference). Here, the results of the current study suggest that IPRPs, as offered in this study, may have beneficial effects on physical functioning and pain in patients with EDS/HSD, although to a somewhat lower degree than for those with a spinal diagnosis. Thus, adapted interventions might be helpful to further improve the benefits of IPRPs for patients with EDS/HSD (20, 35).The results of the current study are in line with previous research demonstrating improvements from outpatient IPRPs on several health measures for patients with EDS/HSD (22). Recent reviews conclude that interventions with a focus on physiotherapy seem to work (18, 35, 36), and that there are no major differences in improvements between patients with EDS and those with HSD (37). Previous studies are generally quite small and researchers have pointed out a need for improved study quality in order to draw reliable conclusions (17). A strength of the current study is thus the large study sample. As a registry-based cohort study (RBCS) it cannot, however, provide the same level of evidence as prospective randomized controlled trials (RCTs). In the absence of a control group not receiving IPRP, we cannot rule out improvements caused by unknown factors. However, the current study can, in some respects, provide more generalizable evidence than a RCT, considering the number of pain clinics, naturalistic recruitment, and the high number of patients with chronic pain included.

One potential weakness of the current study is the absence of possibilities for validation of diagnosis. The current dataset describes the years before the new diagnostic guidelines for EDS and HSD were published in 2017, and it is reasonable to believe that not all physicians involved were sufficiently familiar with EDS/HSD to correctly identify these conditions, and that some patients instead received a less specific pain-related diagnosis. Consequently, the EDS/HSD group in the current study could have been larger, as some patients could instead be categorized under other diagnostic labels. Furthermore, there was a substantial amount of missing data, especially at the 1-year follow-up. Here, only 40% of those with EDS/HSD answered the questionnaire while 51–55% of the patients in the other groups answered. Our pattern mixture-models revealed that missingness was not a major source of concern; however, the missing analysis comes with its own set of assumptions that cannot be verified empirically; therefore the current findings should be interpreted with caution.

Another limitation of this study is that there are large variations in the content and intensity of activities in the IPRPs in different settings (13). Personal knowledge of the healthcare providers, and therefore the possibility to adapt the programmes to specific needs, are also possible confounders of the results.

In conclusion, this study examined differences on key outcome measures of interdisciplinary pain rehabilitation for patients with Ehlers-Danlos Syndrome and hypermobility spectrum disorder, fibromyalgia, spinal pain or whiplash-associated disorders. The results show that the improvements were largely similar among groups, but that patients with a spinal-pain related diagnosis showed a larger improvement on measures of physical functioning, mean pain level for the last week, and pain interference than did patients with EDS/HSD.

## Supplementary Material

INTERDISCIPLINARY PAIN REHABILITATION FOR PATIENTS WITH EHLERS-DANLOS SYNDROME AND HYPERMOBILITY SPECTRUM DISORDERS

## References

[1] Kinge JM, Knudsen AK, Skirbekk V, Vollset SE. Musculoskeletal disorders in Norway: prevalence of chronicity and use of primary and specialist health care services. BMC Musculoskelet Disord 2015; 16: 75. DOI: 10.1186/s12891-015-0536-z 25887763 PMC4392859

[2] Malfait F, Francomano C, Byers P, Belmont J, Berglund B, Black J, et al. The 2017 international classification of the Ehlers–Danlos syndromes. Am J Med Genet C Semin Med Genet 2017; 175: 8–26. DOI: 10.1002/ajmg.c.31552 28306229

[3] Syx D, Wandele ID, Rombaut L, Malfait F. Hypermobility, the Ehlers-Danlos syndromes and chronic pain. Clin Exp Rheumatol 2017; 35: 116–122. 28967365

[4] Castori M, Tinkle B, Levy H, Grahame R, Malfait F, Hakim A. A framework for the classification of joint hypermobility and related conditions. Am J Med Genet C Semin Med Genet 2017; 175: 148–157. DOI: 10.1002/ajmg.c.31539 28145606

[5] Rombaut L, Malfait F, De Paepe A, Rimbaut S, Verbruggen G, De Wandele I, et al. Impairment and impact of pain in female patients with Ehlers-Danlos syndrome: a comparative study with fibromyalgia and rheumatoid arthritis. Arthritis Rheum 2011; 63: 1979–1987. DOI: 10.1002/art.30337 21391202

[6] Castori M. Pain in Ehlers-Danlos syndromes: manifestations, therapeutic strategies and future perspectives. Expert Opin Orphan Drugs 2016; 4: 1145–1158. DOI: 10.1080/21678707.2016.1238302

[7] Baets SD, Cruyt E, Calders P, Dewandele I, Malfait F, Vanderstraeten G, et al. Societal participation in ehlers-danlos syndromes and hypermobility spectrum disorder, compared to fibromyalgia and healthy controls. PLOS One 2022; 17: e0269608. DOI: 10.1371/journal.pone.0269608 35709306 PMC9202833

[8] Molander P, Novo M, Hållstam A, Löfgren M, Stålnacke B-M, Gerdle B. Ehlers–Danlos syndrome and hypermobility syndrome compared with other common chronic pain diagnoses – a study from the Swedish Quality Registry for Pain Rehabilitation. J Clin Med 2020; 9: 2143. DOI: 10.3390/jcm9072143 32645981 PMC7408708

[9] Ringqvist Å, Dragioti E, Björk M, Larsson B, Gerdle B. Moderate and stable pain reductions as a result of interdisciplinary pain rehabilitation-a cohort study from the Swedish Quality Registry for Pain Rehabilitation (SQRP). J Clin Med 2019; 8: 905. DOI: 10.3390/jcm8060905 31238588 PMC6617026

[10] Cohen SP, Vase L, Hooten WM. Chronic pain: an update on burden, best practices, and new advances. The Lancet 2021; 397: 2082–2097. DOI: 10.1016/S0140-6736(21)00393-7 34062143

[11] Gatchel RJ, Peng YB, Peters ML, Fuchs PN, Turk DC. The biopsychosocial approach to chronic pain: Scientific advances and future directions. Psychol Bull 2007; 133: 581–624. DOI: 10.1037/0033-2909.133.4.581 17592957

[12] Gerdle B, Fischer MR, Ringqvist Å. Interdisciplinary Pain Rehabilitation Programs: Evidence and Clinical Real-World Results. IntechOpen; 2022 [cited 2022 Jun 30]. DOI: 10.5772/intechopen.102411

[13] Rivano Fischer M, Schults M-L, Stålnacke B-M, Ekholm J, Persson EB, Löfgren M. Variability in patient characteristics and service provision of interdisciplinary pain rehabilitation: a study using the Swedish national quality registry for pain rehabilitation. J Rehabil Med 2020; 52: jrm00128. DOI: 10.2340/16501977-2765 33191437

[14] Waterschoot FPC, Dijkstra PU, Hollak N, de Vries HJ, Geertzen JHB, Reneman MF. Dose or content? Effectiveness of pain rehabilitation programs for patients with chronic low back pain: a systematic review. Pain 2014; 155: 179–189. DOI: 10.1016/j.pain.2013.10.006 24135435

[15] Kamper SJ, Apeldoorn AT, Chiarotto A, Smeets RJEM, Ostelo RWJG, Guzman J, et al. Multidisciplinary biopsychosocial rehabilitation for chronic low back pain: Cochrane systematic review and meta-analysis. BMJ 2015; 350: h444. DOI: 10.1136/bmj.h444 25694111 PMC4353283

[16] Swedish Council on Health Technology Assessment. Rehabilitation for chronic pain: a systematic literature review. Stockholm: SBU, Swedish Agency for Health Technology Assessment and Assessment of Social Services; 2010. Report no.: 177–1+2.

[17] Corrado B, Ciardi G. Hypermobile Ehlers-Danlos syndrome and rehabilitation: taking stock of evidence based medicine: a systematic review of the literature. J Phys Ther Sci 2018; 30: 843–847. DOI: 10.1589/jpts.30.847 29950777 PMC6016292

[18] Palmer S, Davey I, Oliver L, Preece A, Sowerby L, House S. The effectiveness of conservative interventions for the management of syndromic hypermobility: a systematic literature review. Clin Rheumatol 2021; 40: 1113–1129. DOI: 10.1007/s10067-020-05284-0 32681365 PMC7895781

[19] Baeza-Velasco C, Bulbena A, Polanco-Carrasco R, Jaussaud R. Cognitive, emotional, and behavioral considerations for chronic pain management in the Ehlers-Danlos syndrome hypermobility-type: a narrative review. Disabil Rehabil 2019; 41: 1110–1118. DOI: 10.1080/09638288.2017.1419294 29357706

[20] Chopra P, Tinkle B, Hamonet C, Brock I, Gompel A, Bulbena A, et al. Pain management in the Ehlers-Danlos syndromes. Am J Med Genet C Semin Med Genet 2017; 175: 212–219. DOI: 10.1002/ajmg.c.31554 28186390

[21] Bathen T, Hångmann AB, Hoff M, Øinæs Andersen L, Rand-Hendriksen S. Multidisciplinary treatment of disability in Ehlers-Danlos syndrome hypermobility type/hypermobility syndrome: a pilot study using a combination of physical and cognitive-behavioral therapy on 12 women. Am J Med Genet A 2013; 161A: 3005–3011. DOI: 10.1002/ajmg.a.36060 23913726

[22] Hakimi A, Bergoin C, Mucci P. Immediate and 6-week after effects of a rehabilitation program for Ehlers–Danlos syndrome hypermobile type patients: a retrospective study. Am J Med Genet A 2020; 182: 2263–2271. DOI: 10.1002/ajmg.a.61772 32738018

[23] Rahman A, Daniel C, Grahame R. Efficacy of an out-patient pain management programme for people with joint hypermobility syndrome. Clin Rheumatol 2014; 33: 1665–1669. DOI: 10.1007/s10067-014-2539-9 24557080

[24] Dworkin RH, Turk DC, Farrar JT, Haythornthwaite JA, Jensen MP, Katz NP, et al. Core outcome measures for chronic pain clinical trials: IMMPACT recommendations: Pain 2005; 113: 9–19. DOI: 10.1016/j.pain.2004.09.012 15621359

[25] Kaiser U, Kopkow C, Deckert S, Neustadt K, Jacobi L, Cameron P, et al. Developing a core outcome domain set to assessing effectiveness of interdisciplinary multimodal pain therapy: the VAPAIN consensus statement on core outcome domains. Pain 2018; 159: 673–683. DOI: 10.1097/j.pain.0000000000001129 29300277

[26] Haefeli M, Elfering A. Pain assessment. Eur Spine J 2006; 15: S17–S24. DOI: 10.1007/s00586-005-1044-x 16320034 PMC3454549

[27] Bergström G, Jensen IB, Bodin L, Linton SJ, Nygren Å L, Carlsson SG. Reliability and factor structure of the multidimensional pain inventory – Swedish language version (MPI-S). Pain 1998; 75: 101–110. DOI: 10.1016/S0304-3959(97)00210-8 9539679

[28] Rabin R, Charro F de. EQ-SD: a measure of health status from the EuroQol Group. Ann Med 2001; 33: 337–343. DOI: 10.3109/07853890109002087 11491192

[29] Sullivan M, Karlsson J, Ware JE. The Swedish SF-36 Health Survey-I. Evaluation of data quality, scaling assumptions, reliability and construct validity across general populations in Sweden. Soc Sci Med 1995; 41: 1349–1358. DOI: 10.1016/0277-9536(95)00125-Q 8560302

[30] Hedeker D, Gibbons RD. Longitudinal data analysis. Vol. 2006. Wiley; 2006 [cited 2021 Mar 15]. Available from: https://www.wiley.com/en-us/Longitudinal+Data+Analysis-p-9780471420279

[31] Feingold A. Confidence interval estimation for standardized effect sizes in multilevel and latent growth modeling. J Consult Clin Psychol 2015; 83: 157–168. DOI: 10.1037/a0037721 25181028 PMC4324017

[32] Hedeker D, Gibbons RD. Application of random-effects pattern-mixture models for missing data in longitudinal studies. Psychol Methods 1997; 2: 64–78. DOI: 10.1037/1082-989X.2.1.64

[33] Bates D, Mächler M, Bolker B, Walker S. Fitting Linear Mixed-Effects Models Using lme4. J Stat Softw 2015; 67: 1–48. DOI: 10.18637/jss.v067.i01

[34] Treede R-D, Rief W, Barke A, Aziz Q, Bennett MI, Benoliel R, et al. Chronic pain as a symptom or a disease: the IASP Classification of Chronic Pain for the International Classification of Diseases (ICD-11). Pain 2019; 160: 19–27. DOI: 10.1097/j.pain.0000000000001384 30586067

[35] Reychler G, De Backer M, Piraux E, Poncin W, Caty G. Physical therapy treatment of hypermobile Ehlers–Danlos syndrome: a systematic review. Am J Med Genet A 2021; 185: 2986–2994. DOI: 10.1002/ajmg.a.62393 34145717

[36] Buryk-Iggers S, Mittal N, Santa Mina D, Adams SC, Englesakis M, Rachinsky M, et al. Exercise and rehabilitation in people with Ehlers-Danlos syndrome: a systematic review. Arch Rehabil Res Clin Transl 2022; 4: 100189. DOI: 10.1016/j.arrct.2022.100189 35756986 PMC9214343

[37] Aubry-Rozier B, Schwitzguebel A, Valerio F, Tanniger J, Paquier C, Berna C, et al. Are patients with hypermobile Ehlers–Danlos syndrome or hypermobility spectrum disorder so different? Rheumatol Int 2021; 41: 1785–1794. DOI: 10.1007/s00296-021-04968-3 34398260 PMC8390400

