# Supplementary material for: INTERDISCIPLINARY PAIN REHABILITATION FOR PATIENTS WITH EHLERS-DANLOS SYNDROME AND HYPERMOBILITY SPECTRUM DISORDERS
Source: J Rehabil Med. 2024 Feb 7;56:12431. doi: 10.2340/jrm.v56.12431 (PMC10863620; doi:10.2340/jrm.v56.12431)
Supplement: Supplementary file 1 — INTERDISCIPLINARY PAIN REHABILITATION FOR PATIENTS WITH EHLERS-DANLOS SYNDROME AND HYPERMOBILITY SPECTRUM DISORDERS [file JRM-56-12431-s1.pdf]

Supplementary material has been published as submitted. It has not been copyedited, typeset or checked for scientific content by Journal of Rehabilitation Medicine

Table SI. Differences between follow-up estimated values with standard errors for ordinary growth models and growth models including missing pattern analysis

Diagnosis	EDS/HSD	WAD	Spinal	FMS
Base model	53.76 (1.36)	63.91 (0.9)	63.79 (0.42)	53.61 (0.48)
With missing pattern	52.4 (1.55)	64.6 (1.18)	65.3 (0.53)	55.3 (0.63)
Difference	-1.36	0.69	1.51	1.69

There was also a difference on MPI Pain Interference of 0.02 scores for the EDS-group, where the mean value at follow-up before the missing pattern analysis was 3.87, and after 3.85. For those with FMS, taking missingness into account gave a follow-up value of 3.73, and with an uncorrected value of 3.93. The effect size of the difference between these groups is then 0.09.

EDS/HSD: Ehlers-Danlos Syndrome/Hypermobility Syndrome; WAD: whiplash related diagnosis; FMS: fibromyalgia syndrome.
